# Heart Rate Variability in Sport Performance: Do Time of Day and Chronotype Play A Role?

**DOI:** 10.3390/jcm8050723

**Published:** 2019-05-21

**Authors:** Jacopo Antonino Vitale, Matteo Bonato, Antonio La Torre, Giuseppe Banfi

**Affiliations:** 1 IRCCS Istituto Ortopedico Galeazzi, Via Riccardo Galeazzi 4, 20161 Milan, Italy; matteo.bonato@unimi.it (M.B.); antonio.latorre@unimi.it (A.L.T.); banfi.giuseppe@hsr.it (G.B.); 2 Department of Biomedical Sciences for Health, Università degli Studi di Milano, Via Giuseppe Colombo 71, 20133 Milan, Italy; 3 Vita-Salute San Raffaele University, Via Olgettina 58, 20132 Milan, Italy

**Keywords:** HRV, circadian typology, physical activity, orthopaedics, HIIT.

## Abstract

A reliable non-invasive method to assess autonomic nervous system activity involves the evaluation of the time course of heart rate variability (HRV). HRV may vary in accordance with the degree and duration of training, and the circadian fluctuation of this variable is crucial for human health since the heart adapts to the needs of different activity levels during sleep phases or in the daytime. In the present review, time-of-day and chronotype effect on HRV in response to acute sessions of physical activity are discussed. Results are sparse and controversial; however, it seems that evening-type subjects have a higher perturbation of the autonomic nervous system (ANS), with slowed vagal reactivation and higher heart rate values in response to morning exercise than morning types. Conversely, both chronotype categories showed similar ANS activity during evening physical tasks, suggesting that this time of day seems to perturb the HRV circadian rhythm to a lesser extent. The control for chronotype and time-of-day effect represents a key strategy for individual training schedules, and, in perspective, for primary injury prevention.

## 1. Introduction

The way in which the cardiovascular system responds to exercise stress has captured the imagination of sport scientists over the past century [1]. During physical activity, critical adjustments are continually made by the cardiovascular system to meet the diver’s demands with respect to the musculature and the heart [2]. These dynamic adjustments in cardiac and peripheral vascular control, including their regulation by the autonomic nervous system (ANS), occur in response to rapid changes in the heart rate (HR) and blood pressure. These variations also include circadian variations during the course of the day, which could have either a positive or negative effect on sport performance [3]. For this reason, understanding the interactions between cardiovascular function, activity of the ANS, chronobiology, biological rhythms, and chronotype allows to us understand the effects of exercise on human performance. Therefore, the aim of this narrative review is to discuss the circadian pattern of heart rate variability (HRV) and the effect of time-of-day and chronotype on HRV circadian rhythm in response to acute physical activity

## 2. Chronobiology, Biological Rhythms, and Chronotype

Chronobiology (from three Greek words: “kronos” for time, “bios” for life, and “logos” for study) is the science that objectively studies the biological mechanisms of time structures. Rhythms can be observed at all levels of biologic integration, and show different frequencies: (1) the ultradian rhythm (<20 h; e.g., a full sleep cycle) [4]; (2) circadian rhythm (period between 20 h and 28 h, specifically relating to biological variations over 24 hours [5,6,7]); and (3) the infradian rhythms (period >28 h, including circaseptan, circadiseptan, circavigintan, circatrigintan, and circannual rhythms [5,8,9,10]). Each biological rhythm has specific quantifiable characteristics and usually three different parameters are described: (1) acrophase (Ø), which indicates the time interval within which the highest values are observed; (2) amplitude (A), a measure of one half of the extent of rhythmic variation in a cycle; and (3) MESOR, acronym of Midline Estimating Statistic of Rhythm (M), the rhythm-determined mean [10,11]. The existence of human’s circadian rhythms is explained by the interaction of several multifactorial systems cooperating at the same time, including exogenous, endogenous, and lifestyle mechanisms [12]. Specifically, the internal master circadian clock resides within the suprachiasmatic nuclei (SCN) of the anterior hypothalamus [13] and autonomous circadian clocks are present in nearly all tissues [14]. On the other hand, the SCN is also synchronized with the environment by external factors called “*zeitgebers*” (“time-givers” in German) or “synchronizers”. The primary synchronizer for the human body clock is the light–dark cycle; the light gives information to the SCN, passing from retinal ganglion cells via a direct pathway (the retinohypothalamic tract), leading to a synchronization of the peripheral clocks by neuro-normal signaling [15,16]. In addition, many other variables could play the role of secondary synchronizers, such as physical activity, sleep and meal timing, and/or social routine [6,16]; it has indeed been shown that regular training, intended as chronic exercise, is associated with better nocturnal sleep and with a physiological circadian expression of steroid hormones in healthy and pathological conditions [6,17]. In general, the correct expression of biological rhythms is crucial for body homeostasis since individuals perform optimally when all biological rhythms are in sync [18].

Nevertheless, it is important to highlight that circadian rhythmic expression may largely vary among individuals, and this characteristic is typically defined as “circadian typology” or “chronotype”. The chronotype is usually evaluated using self-assessment questionnaires, validated in several forms and countries [19,20,21,22,23], and the most-used and cited questionnaire is the Morningness–Eveningness Questionnaire (MEQ) [24]. There are three different chronotypes—morning types (M-types or “larks”), evening types (E-types or “owls”), and neither types (N-types) —that represent an individual’s predisposition towards morningness or eveningness [19]. The chronotype distribution is influenced both by environmental (i.e., latitude and photoperiod at birth) and individual factors, such as sex and age: men are typically E-types while women tend to be M-types, especially before 40 years of age; however, this trend is overturned with advancing age [25], and in general, after the end of adolescence, morningness scores tend to increase with age [26]. Chronotype does not concern just a subjective predisposition; indeed, several studies have shown large bio-psycho-physiological differences between M-types and E-types in relation to the circadian rhythms of different variables. M-types, for instance, use to wake up and go to bed earlier than E-types [27], both during weekend and week days [28], and they display an advanced acrophase of blood melatonin concentrations and rest–activity circadian rhythm by about 02:30 h [19,28]. On the other hand, E-types show delayed acrophases of oral temperature (+2 hours) and serum cortisol (+55 minutes) circadian rhythms as compared to M-types [29,30]. It is also important to note that chronotype can also affect human cognitive and physical performance [19,31]. It was shown that M-types have greater vigor levels and higher memory task scores in the morning compared to E-types [31,32] and, in addition, morning-oriented subjects registered faster race times in the morning for the half marathon, full marathon, and 200-m swimming trial than the other chronotypes [33,34]. Conversely, people with a strong predisposition toward eveningness reach their best performances later in the day: E-types seem to have more of an advantage and to be less fatigued in the second part of the day than N- and M-types [35,36,37,38]. Therefore, it seems that the chronotype could play a key role in determining the circadian expression of the human body clock in different conditions and settings.

## 3. HRV Assessment

The HR and circulatory systems are controlled primarily by higher brain centers and cardiovascular control areas in the brain stem through the activity of the ANS, which is composed of sympathetic and parasympathetic nerves. The medulla is the primary site to regulate sympathetic and parasympathetic (vagal) outflow to the heart and blood vessels [39]. The rate and variation of heart beats are the results of a complex interaction between sympathetic and parasympathetic efferent impulse activity in addition to the influence of sinus node pacemaker properties [40]. The sinoatrial (SA) node is directly and richly innervated by both sympathetic and parasympathetic (vagus) nerve fibers, which are continually active; the atrioventricular (AV) node is less affected. Parasympathetic stimulation hyperpolarizes the SA node, decreasing the rate of spontaneous firing and the cardiac rate. On the other hand, sympathetic nerve endings release norepinephrine and the adrenal medulla releases epinephrine, stimulating the spontaneous firing rate of the SA and increasing cardiac rate. Although both the sympathetic and parasympathetic systems are active at rest, the parasympathetic fibers release acetylcholine, which acts to slow the pacemaker potential of the SA node and thus reduces heart rate [39]. Therefore, sympathetic stimulation increases the HR, contractility, and conduction velocity, whereas parasympathetic stimulation has the opposite effect. In addition, autonomic control of the cardiovascular system is also affected by baroreceptors, chemoreceptors, muscle afferents, local tissue metabolism, and circulating hormones [1].

Since the ANS is linked with many other physiological systems, its responsiveness may provide useful information about the functional adaptations of the body during and after exercise [1]. One of the most non-invasive reliable methods to assess the ANS activity is to evaluate the time course of heart rate variability (HRV), which is the natural fluctuation of HR in time due to internal and external body process [1]. It is usually measured as the standard (or average) deviation from the mean intervals between successive heartbeats (NN intervals or R-R intervals) of all cardiac cycle lengths (R-R intervals for normal sinus beats) over a given period, from 5 minutes to 24 hours [41]. Originally, HRV was assessed manually from calculations of the mean R-R interval and its standard deviation measured on a short-term electrocardiogram (ECG). Recently advances in recording techniques and innovative smart devices have enabled the quantification of autonomic functions. Several algebraic methods and graphs allow us to study and describe the HRV, among which the most used correspond to the methods of the time domain, in which the R-R intervals (in milliseconds) are plotted against time (in seconds) and frequency domain, measuring the frequency at which the length of the R-R intervals changes. Another measurement is the standard deviation of the normal R-R interval, known as the SDNN (ms) index. This measure basically shows how much the HR differs from the overall daylong mean HR. Yet others have used the standard deviation (SD) index, that is, the mean of the SD computed for each successive 5-min period over 24-h, measuring the variation that occurs within 5-min periods rather than the variation that occurs over longer time intervals. Other measures include pNN50 index (%), which shows the instances per hour in which two consecutive normal R-R intervals differ by more than 50 ms over 24-h; the base of the triangular area under the main peak of the R-R interval frequency distribution diagram obtained from 24-h recording; and the RMSSD (ms) index, the root-mean square of the difference of successive R-R intervals. Although it is generally accepted that the various methods measuring peak-to-peak variation in cardiac cycle length can be used as an index of parasympathetic activity, information on the changes in both sympathetic and parasympathetic activity of the heart may be obtained only by using spectral analysis. The peak-to-peak variation is usually represented as a tachogram in which the signals sampled at regular are interpolated and synchronized with the QRS complexes of the ECG [1]. Both methods basically measure a random signal. Spectral analysis of the HRV provides information on the different statistical components of the signal. It transforms the signal from time to frequency on the x-axis by representing it as a combination of sine and cosine waves, with different amplitudes and frequencies which are used to describe its spectral components. This is the classic nonparametric approach for determining rhythmic components and is known as the Fast Fourier transform (FFT). The FFT is an objective method in which the tachogram provides a spectrum of the high frequency power (HF), which is defined by the energy in the HR power spectrum between 0.15 and 0.40 Hz, evaluating the parasympathetic activity, and the low frequency power (LF), defined by the energy in the HR spectrum between 0.04 to 0.15 Hz, which defines both sympathetic and parasympathetic activity [1]. Ultra-low frequency power (ULF, ≤0.003 Hz) and very low frequency power (VLF, 0.003–0.04 Hz) can also be measured [1].

However, the HR is continuously modulated by non-linear fluctuations due to postural changes, physical activity, and multiple interactions with other physiological systems, and it may also be affected by small perturbations (e.g. premature ventricular contractions, atrioventricular block) [42,43]. These measurements involve the quantification of the “chaos” in heart rhythms or the behavior of HRV patterns over different time scales (i.e., a few minutes vs. 24 hours). The most common non-linear methods applied to HRV are: (1) the Poicarè plot [44]; (2) approximate entropy [45]; (3) sample entropy [46]; (4) correlation dimension [47]; and (5) detrended fluctuation analysis [48] and recurrence plots [49]. Despite the higher computational complexity required, these approaches have recently proven superior in quantifying and mapping non-linear and chaotic ANS activities, as well as in correlating HRV signals to precise psychological states of research subjects [50]. When applying these methods is important to ensure low signal-to-noise ratio, accurate estimation of high-frequency spectrum in short-time recordings, and low variability of signals [51].

It has also been demonstrated that HRV can be affected by respiration frequency [1,39,41]. Generally, the HRV increases when respiratory frequency decreases. Although respiration greatly affects the HRV, the absence of standardized models of respiratory frequency makes it difficult to interpret HRV data. According to previous studies, researchers have accepted various respiratory frequency ranges (e.g. from 6 to 15 breath/min). However, it is clear that a self-organized respiratory pattern should be maintained during the recording period [1,39]. Also, it is important that standard protocols and methods be established with athletes with regard to exercise intensity and duration, respiration rate, position of the body during recording, and duration of recording [1].

## 4. HRV Circadian Rhythm

Among all the physiological function showing a circadian rhythmicity, the cardiovascular system displays a marked daily rhythm in most of the physiological parameters, including HR and blood pressure [52]. HR circadian rhythm starts to raise around awakening time or soon after the beginning of the individual’s activity; it reaches the acrophase between 10:00 and 12:00 h and maintains a lower level during the night [53]. This HR fluctuation is crucial for human health since it guarantees that the heart adapts to the needs of different activity levels during sleep phases or daytime by decreasing or increasing the cardiac output [53,54]; as a consequence, HRV shows marked circadian variations. These daily fluctuations of the ANS [55,56] that reflect the sympathovagal balance activity are commonly found at rest in healthy subjects [57]. In general, HRV parameters tend to increase during the nighttime and to decrease during the day, showing however a larger variability around awakening when HR changes rather abruptly from the nightly low to the much higher daily values [55,56]. The actual transition usually starts earlier, but any anticipatory rise preceding awakening and the transition in the evening is smoother and slower [58,59]. It has also been observed that age is able to affect the HRV circadian rhythm. Due to the immaturity of the ANS and the increased sleep time, children under 12 months old do not register a significant HRV rhythm [58]. On the contrary, increasing age corresponds to a reduced power of the 24-hour HRV [60], a decline in efferent vagal cardiac tone, and a decreased beta-adrenergic responsiveness [61,62,63,64]. Autonomic derailments have also been reported to augment cardiovascular degeneration in the aging population, shifting the autonomic balance toward sympathetic dominance [65,66,67]. Some pathological conditions (i.e., ischemic cardiac disease) are able to affect the ANS function with the amplitude of the HRV circadian rhythm that can be altered, flattened, or nearly absent [59,60,68,69], with altered acrophases and MESOR values. Indeed, the lowest HRV nadir that is observed in the morning [62] corresponds to the period of highest incidence of ventricular tachycardia and sudden death [70,71,72]. This unhealthy circadian pattern has been already observed in patients affected by other pathological conditions, such as diabetes, obesity, metabolic syndrome, and cancer [7], and this further confirms the need to maintain and correct the circadian system. For these reasons, it is reasonable to believe that HRV circadian rhythm is linked to wake time and daily physical activity (PA) levels, although results are still sparse and this question has not been fully studied. In line with this hypothesis, it has been shown that there are a series of changes in the cardiovascular system soon after waking and commencing activity, which include an increase in HR, blood pressure, plasma catecholamine levels, and renin activities [73]. In the next chapter, we will discuss the effect of time-of-day and chronotype on HRV circadian rhythm in response to acute PA.

The changes in HRV have also been studied to explore sympathovagal balance during sleep [74]. It has been observed that slow-wave sleep is characterized by decreased LF with a relative predominance of HF as compared to a waking state [75]. Moreover, it was suggested that rapid eye movement sleep is characterized by an HRV pattern with increased linear variability as compared to slow-wave sleep and wakefulness [75]. In addition, Vigo et al., [76] demonstrated that state slow-wave sleep is characterized with increased HRV, whereas rapid eyes movements (REM) sleep is associated with increased linear HRV in all frequency components. Specifically, they observed that during the slow-wave cycle, HRV was characterized by high-entropy VLF and increased relative amplitude in the HF component with rapid eye movement sleep that were indistinguishable from the wake phase with respect to nonlinear HRV, and were associated with increased linear HRV globally and all its frequency components.

## 5. HRV and Physical Exercise

During physical exercise the vagal tone is withdrawn and HR is regulated principally by adrenergic activity [77]. The mechanism of the exercise-induced tachycardia involves parasympathetic and spinal sympathetic reflex circuits. Thus, both the sympathetic and parasympathetic arms of the ANS play a pivotal role during exercise. Changes in HRV in response to exercise can vary in accordance to the degree and duration of training and/or the kind of training [78,79]. The HR increase during exercise is due both a parasympathetic withdrawal and an augmented sympathetic activity, and the relative role of the two drivers depends on exercise intensity. In particular, changes in HRV, including significant variations in LF, HF, and total power of the frequency domain, were observed in response to different intensities of aerobic exercise. The HF peak is recognized in the power spectrum in the full range of relative intensity, being responsible for the most part of HRV at maximal load, while LF power does not change during low intensity exercise (below anaerobic threshold) and usually decreases to negligible values at medium–high intensity (above anaerobic threshold), where sympathetic activity is enhanced [79]. According to this, it has been observed that the increase in HR during exercise is coupled with the decrease in HRV in both HF and LF frequencies during a graded-work load exercise on a cycle ergometer [80], and the largest decrease in HRV total power has been reported to occur during steady state exercises [81,82]. In general, it seems that the changes in HF and LF power observed during exercise do not reflect the decrease in vagal activity and the activation of sympathetic system occurring at increasing loads [39]. Furthermore, during exercise, technical problems arise from HR measurements because the steady state, which is necessary for spectral analysis, is not always obtained [1,77,78]. The HRV measurement immediately after the cessation of exercise reflects the subjects’ responses to exercise, which primarily correlates to physical fitness. In this regard, Hautala et al. [83] observed that the parasympathetic tone was reduced after prolonged exercise and demonstrated that the recovery time was inversely correlated with subjects’ maximal aerobic power. The effects of exercise on nocturnal HRV were also investigated [84]. In this study, 14 male subjects performed five different running exercises on a treadmill with the aim of studying the effects of exercise duration (30, 60, 90 minutes) and intensity (45%, 60%, 75% maximal oxygen uptake), and the results suggested that the increasing exercise intensity and/or duration caused a delayed recovery of nocturnal cardiac autonomic modulation [84]. Regarding the effects of supramaximal intermittent exercise, it was observed that there is a strong influence of exercise intensity on short- and long-term HRV recovery [85], because it improves post-exercise parasympathetic function [86]. In general, it has been demonstrated that athletes have, both at rest and after exercise, lower HR values than sedentary controls [2]. To confirm this, Imai et al. [87] observed that vagal-mediated HR recovery after 30 and 120 seconds after exercise performed at different intensities (i.e., maximal exercise, at anaerobic threshold, at 50% of anaerobic threshold) was accelerated in well-trained athletes, highlighting a physiologic adaptation allowing for rapid HR recovery after intense exercise. For this reason, according to the meta-analysis of Bosquet et al. [88], HRV has been widely researched in elite and well-trained athletes in order to understand the body’s response via the ANS to intensified training loads. A potential role on monitoring ANS homeostasis via HRV to aid in overuse injury detection was also hypothesized [89]. In fact, considering the complex interactions of external and internal factors involved in overuse injury development, authors hypothesized that athletes with accumulating somatic tissue damage would demonstrate HRV modulations at rest, reflecting decreased parasympathetic control and increased SNS response. Relative to each athlete’s baseline HRV measurements, imbalances in parasympathetic nervous system and sympathetic nervous system activity may indicate that an athlete is in a state of ongoing repair and recovery, as compared to an athlete who is adapting positively to training load [89]. Table 1 summarizes the interventional studies assessing the effects of physical activity on HRV.

## 6. Time-of-Day and Chronotype Effect on HRV in Response to Acute PA

The different timing of physical exercise could potentially facilitate phase shifts of the human circadian rhythms (e.g. HR and HRV circadian rhythms) to a new time schedule, hence suggesting a “synchronizing” effect of physical activity [6,90,91]. However, few studies adopted a chronobiological approach to evaluate the effect of morning and/or evening exercise on HRV. A recent study by Yamanaka and colleagues [92] evaluated the effect of morning and evening physical exercise on the ANS during sleep phases in 22 healthy male subjects. HRV significantly changed and it was observed that VLF, LF, and HF waves increased after morning exercise, while the subjects’ HR increased after evening exercise. The authors concluded that imposed exercise of 2 hours consisting of cycling or rowing imposed in the morning enhanced the parasympathetic activity, while in the evening, exercise increased the sympathetic activity during the night [92]. Another study tested the diurnal changes in HRV 24-h pre- and post-maximal aerobic exercise testing to exhaustion in both middle-aged and young adults [93]. It was reported that young subjects had higher HRV (i.e., higher HF power) compared to middle-aged adults during morning, afternoon, evening, and night prior to exercise, and furthermore, exercise resulted in reductions in HRV during the afternoon and evening periods [93]. Similarly, Prodel et al. [94] tested the impact of 35 minutes of cycling exercise performed at the first anaerobic threshold, at three times of day (7:00, 14:00, and 23:00 h), on HR and HRV during sleep in nine healthy sedentary subjects. As expected, exercise determined an increase in HR and a decrease in HRV but different times of day did not affect the magnitude of this difference. In addition, exercise performed at 07:00 h did not delay exercise recovery; HR was similar to rest after 15 minutes of recovery, and HRV was similar to rest after 30 minutes of recovery in the morning, afternoon, and night. Morning exercise did not delay HR and HRV recovery after light aerobic cycling exercise and the authors concluded that sedentary subjects could engage physical activity at any time of day without higher risk [93].

Nonetheless, to the best of our knowledge, only a small number of studies evaluated the chronotype as a factor able to influence HRV circadian rhythm in response to morning or evening acute exercise. In this regard, Nebel et al. [95] studied the effect of mental and physical stressors in the morning and at noon in M- and E-types and it was observed that morning-oriented subjects had higher HR and rate by pressure product (RPP: HR × systolic blood pressure) during the morning session, while E-types exhibited higher levels during the afternoon [95]. Similarly, it was shown that E-types had significantly higher HR and RPP at 14:00 h than at 09:00 h at rest and in response to a battery of three stress tasks [96] and that E-type students registered lower absolute levels of HRV and higher levels of HR than M-types in response to a mental arithmetic task, especially in the evening [97]. Another study conducted by Sugawara et al. [98] tested the effect of chronotype on post-exercise vagal reactivation, evaluated by the monitoring of the time constants of the beat-by-beat HR decay for the first 30 s after exercise, in 37 students classified as N-types (*n* = 23), M-types (*n* = 6) and E-types (*n* = 8). The subjects performed two constant-load cycle tests, both in the morning and in the late afternoon, and a significant interaction between chronotype and time was observed for HR recovery: specifically, E-types had a larger morning HR recovery than both M-types (165.5 ± 45.2 vs. 94.4 ± 33.8 s) and their own evening data (119.5 ± 25.7 s). It was therefore stated that post-vagal reactivation was sluggish at 19:00 h for E-types [98]. Recently, Bonato et al. [99] evaluated the chronotype effect on the autonomic cardiac control of 24 soccer players, classified in 12 M-types and 12 E-types, in response to an acute session of high-intensity interval training (HIIT) performed at 08:00 h and 20:00 h. Given that high-intensity intermittent exercise is able to perturb the ANS for more than 24 hours [85], the authors decided to calculate HRV vagal and sympatho/vagal indices in terms of time, frequency, and complexity domains at rest, before and after 12 and 24 hours of HIIT sessions. Before evening HIIT, a higher resting HR was observed for both groups, while E-type players presented significantly higher HR values that corresponded to a lower parasympathetic tone before and after the morning HIIT sessions than M-types; on the contrary, M-type subjects did not reveal any significant differences with E-types during the evening HIIT [99]. In general, these results suggest that an individual’s chronotype should be considered when scheduling a physical training and understanding the post-exercise HRV response seems necessary to monitor the recovery process. Table 2 summarizes the interventional studies assessing the effects of time-of-day and chronotype on HRV in response to acute physical activity.

## 7. Limitations

The discrepancies in the results concerning extremely high interindividual variability of HRV with the psychophysiological responses to morning or evening physical activity in different chronotypes could be explained by several confounding factors and/or methodological issues: (1) most of the discussed studies did not perform matching with regard to age- and sex, and the participants showed different baseline physical conditions; (2) not all chronotypes were represented and the times of day selected for the physical tests widely differed among studies (e.g., extreme vs. moderate times of the day); (3) the choice of the type, duration, and intensity of physical activity was not always adequately controlled; and (4) the habitual training time was not considered when evaluating HRV data, even if this variable could potentially influence the subject’s chronotype, the individual circadian rhythmicity, and the physiological responses to a physical test [6,33,100,101].

## 8. Practical Applications

According to the different studies presented, this review provides practical applications for training management for strength and conditioning; coaches should be aware that M-types and E-types respond in different ways to morning or evening training. Since E-types seems to have a higher perturbation of ANS in the first part of the day than M-types, and, specifically, a slowed vagal reactivation and higher HR values were observed post morning exercise in evening-oriented individuals than morning-oriented subjects, the controls for chronotype and time-of-day effect represent a key strategy for individual training schedules and for primary prevention of muscle and bone injuries. These results are confirmed by previous studies that highlighted that chronotype influences ratings of perceived exertion and physical tasks performed in the morning, where M-types seem to have more of an advantage because they are less fatigued and register greater physical performances than N- and E-types [3]. Conversely, both chronotype categories showed similar ANS activity during evening physical tasks, suggesting that this time of day seems to perturb to a lower extent the HRV circadian rhythm. However, it is important to highlight that the latter statement should be limited to the HRV daily rhythm since many previous studies demonstrated that also late-evening or night physical performances are able to affect other psychophysiological variables (e.g. salivary cortisol or sleep–wake cycle) in athletes or young healthy subjects with different chronotypes [99,101,102].

## 9. Conclusions

Based on the present data, we can conclude that acute physical activity causes a perturbation in the HRV circadian pattern, and consequently, this variable could be influenced by both chronotype and time of the day. Future studies should strongly consider strict protocols such as free running, forced desynchronization, modified sleep times, or constant routine protocols since many confounders could significantly influence sports performances and the circadian rhythms of many biological variables related to exercise.

## Figures and Tables

**Table 1:** Interventional studies that assessed the effects of physical activity on HRV.

Study	Participants	Intervention	Outcome Measure
Arai et al. [80]	43 healthy subjects 8 patients with severe congestive heart failure 6 patients with post cardiac transplantation	Graded-work load exercise on a cycle ergometer.	↓ of autonomic modulation of HR in patients with CVD ↑ vagal activity during exercise and recovery in healthy subjects
Baselli et al. [81]	9 sedentary males 8 professional cyclists	Muscular exercises at different intensities on a cycle ergometer.	↓ of LF power and ↑ of VLF power at the higher exercise level
Gronwald et al. [82]	16 well-trained cyclists	Interval sessions with active recovery periods.	↓ in the overall variability ↓ in the complexity of the R-R interval fluctuations
Hautala et al. [83]	10 cross country skiers	75-km cross-country skiing race.	Cardiac vagal outflow blunted for several hours after the race. The recovery time of reduced vagal outflow depends on athlete cardiorespiratory fitness. ↑ of altered autonomic modulation during the second day after the race
Myllymäki et al. [84]	14 male healthy subjects	5 different running exercises on separate occasions starting at 18:00 h.	↑ exercise intensity and/or duration cause delayed recovery of nocturnal cardiac autonomic modulation.
Al Haddad et al. [85]	11 healthy subjects	Series of two consecutive intermittent 15-s runs at 95% MAV interspersed with 15 s of active recovery at 45% MAV until exhaustion	Influence of exercise intensity on short- and long-term post exercise HRV recovery
Bonato et al. [86]	14 healthy subjects	5 × 30 m sprints with 25-s recovery	↑ post-exercise parasympathetic function Short-term reliability of post-exercise parasympathetic reactivation indices showed large discrepancies in markers of reliability.
Imai, et al. [87]	8 healthy subjects 20 patients with CVD 9 cross-country skiers	HR decay for the first 30 s and the first 120 s after six levels of exercise	Vagally mediated HR recovery after exercise is accelerated in well trained athletes but blunted in patients with chronic heart failure

Legend. HR: heart rate; CVD: cardiovascular diseases; LF: low frequencies; VLF: very low frequencies; MAV: maximal aerobic velocity; HRV: heart rate variability.

**Table 2:** Interventional studies that assessed the effects of time-of-day and chronotype on HRV in response to acute physical activity.

Study	Participants	Intervention	Outcome Measure
Yamanaka et al. [92]	22 healthy male subjects	2-h intermittent physical exercise on cycle ergometer at different times of the day for four consecutive days	↑ of parasympathetic activity after morning exercise ↓ of sympathetic activity during evening exercise
Armstrong et al. [93]	12 young adults and 12 middle-aged healthy subjects	Maximal aerobic capacity test	The change in HRV from sleep to morning with exercise is greater in younger subjects.
Prodel et al. [94]	9 sedentary healthy males	35 min of cycling exercise, at an intensity of first anaerobic threshold, at 07:00 h, 14:00 h, and 23:00 h	Morning exercise did not delay HR and HRV recovery after light aerobic cycling exercise. Exercise performed in the night changed autonomic control during sleep.
Sugawara et al. [98]	6 M-type and 6 E-type healthy male subjects	3-min exercise on cycle ergometer at 80% VT performed in the morning (07:00–08:00 h and in the evening (17:00–18:00 h)	Diurnal variation in post-exercise vagal reactivation is different between the morning-type and evening-type
Bonato et al. [99]	6 M-type and 6 E-type soccer players	Four bouts of 4 minutes at 90–95% HR_peak_ with 3 min of active recovery at 50–60% HR_peak_ performed in the morning (08:00 h) or in the evening (20:00 h)	E-types showed lower parasympathetic tone that returned to the rest values after 24 hours of the cessation of exercise

Legend. HR: heart rate; HRV: heart rate variability; M-types: morning-type; E-type: evening-type; VT: ventilatory threshold.
